# A Computational Text Mining-Guided Meta-Analysis Approach to Identify Potential Xerostomia Drug Targets

**DOI:** 10.3390/jcm11051442

**Published:** 2022-03-05

**Authors:** Micaela F. Beckman, Elizabeth J. Brennan, Chika K. Igba, Michael T. Brennan, Farah B. Mougeot, Jean-Luc C. Mougeot

**Affiliations:** Department of Oral Medicine, Carolinas Medical Center, Atrium Health, Charlotte, NC 28203, USA; micaela.beckman@atriumhealth.org (M.F.B.); liz.brennan.3@gmail.com (E.J.B.); chikaigba7@gmail.com (C.K.I.); mike.brennan@atriumhealth.org (M.T.B.)

**Keywords:** xerostomia, dry mouth, protein–protein interaction, drug target, head and neck cancer, Sjögren’s Syndrome

## Abstract

Xerostomia (subjective complaint of dry mouth) is commonly associated with salivary gland hypofunction. Molecular mechanisms associated with xerostomia pathobiology are poorly understood, thus hampering drug development. Our objectives were to (i) use text-mining tools to investigate xerostomia and dry mouth concepts, (ii) identify associated molecular interactions involving genes as candidate drug targets, and (iii) determine how drugs currently used in clinical trials may impact these genes and associated pathways. PubMed and PubMed Central were used to identify search terms associated with xerostomia and/or dry mouth. Search terms were queried in pubmed2ensembl. Protein–protein interaction (PPI) networks were determined using the gene/protein network visualization program search tool for recurring instances of neighboring genes (STRING). A similar program, Cytoscape, was used to determine PPIs of overlapping gene sets. The drug–gene interaction database (DGIdb) and the clinicaltrials.gov database were used to identify potential drug targets from the xerostomia/dry mouth PPI gene set. We identified 64 search terms in common between xerostomia and dry mouth. STRING confirmed PPIs between identified genes (CL = 0.90). Cytoscape analysis determined 58 shared genes, with cytokine–cytokine receptor interaction representing the most significant pathway (*p* = 1.29 × 10^−23^) found in the Kyoto encyclopedia of genes and genomes (KEGG). Fifty-four genes in common had drug interactions, per DGIdb analysis. Eighteen drugs, targeting the xerostomia/dry mouth PPI network, have been evaluated for xerostomia, head and neck cancer oral complications, and Sjögren’s Syndrome. The PPI network genes IL6R, EGFR, NFKB1, MPO, and TNFSF13B constitute a possible biomarker signature of xerostomia. Validation of the candidate biomarkers is necessary to better stratify patients at the genetic and molecular levels to facilitate drug development or to monitor response to treatment.

## 1. Introduction

### 1.1. Epidemiology and Symptomatology

Xerostomia, also known as the sensation or subjective complaint of dry mouth, is a common condition affecting the oral cavity, mainly due to functional and structural damage to the salivary glands [1]. Prevalence varies and is highly dependent on the population studied and methodologies implemented [2,3,4]. People most significantly impacted include women who are menopausal and individuals over 65 years of age [5,6]. However, younger individuals can also be affected, since approximately 20% of those who report this problem are between the ages of 18 to 34 [5]. Marcott et al. has recently suggested that the condition affects 10% to 46% of people amongst the US, Mexico, and several countries in Europe [7]. A study that analyzed population-based measures of the condition concluded that the prevalence of xerostomia ranges from 9.7 to 25.8% in men and 10.3 to 33.3% in women [8]. 

Symptoms of xerostomia vary physically and can result in general psychological distress [9]. Symptoms often include the feeling of dryness and stickiness of the mouth, difficulty swallowing, chewing or speaking, and having a dry tongue or grooved appearance of the tongue [9]. Patients can also experience sore throat and an altered sense of taste [10]. Because saliva acts to remove excess bacteria and plaque from the teeth, the lack of saliva in xerostomic patients can increase the risk of dental caries and infections [8]. In addition, the subjective perception of xerostomia is frequently, but not always, associated with reduced salivary flow [1,8,11].

### 1.2. Etiologies, Pathophysiology, and Treatment

Xerostomia etiologies include genetic predispositions (i.e., single-nucleotide polymorphisms associated with adverse drug reactions or salivary gland disorders), side effects from medications, radiation treatments to the head and neck, and certain chronic autoimmune diseases [3,5,12,13,14]. Most notably, dry mouth is commonly reported in patients suffering from Sjögren’s Syndrome, rheumatoid arthritis (RA), chronic juvenile arthritis, sarcoidosis, and systemic sclerosis [5].

Current treatments include products to stimulate saliva, mucosal comfort agents, and/or saliva substitutes. These medications address the symptoms of xerostomia but are unable to address the underlying biological mechanisms of the condition [5,15]. A recent study showed that 23.2% of patients in a xerostomia cohort used saliva substitutes, yet the effectiveness of such treatments remained unclear [7]. Vissink et al. found that saliva substitutes can provide temporary relief of xerostomia symptoms [16]. However, a Cochrane review did not find a significant difference in the effectiveness of oral lozenges, sprays, gels, or other saliva substitutes when compared to a placebo [17]. The lack of targeted treatments available contributes to the diminished quality of life of those living with this condition [7,8,15]. 

### 1.3. Data Mining and Objectives

A comprehensive knowledge-based resource detailing the pathways and biological mechanisms of dry mouth pathophysiology could be beneficial to drug target discovery in xerostomia therapeutic development. Advancements in biomedical research over the past decade have led to a significant increase in the number of publications and availability of open access research articles [18]. Data-mining and text-mining tools allow for the sorting and identification of information from a massive amount of research [19]. Many publications introduce individual approaches, yet fewer combine multiple methods to identify genes or expression pathways of interest [20].

Our objective was to utilize a combined data-mining approach to determine curated gene and protein interaction profiles of xerostomia and/or dry mouth to identify potential drugs that can target disease relevant molecular pathways. Our aims were to (i) perform literature mining for the identification of genetic and proteomic profiles associated with xerostomia, (ii) establish the relationship of the concepts “xerostomia” and “dry mouth” with genes, (iii) characterize xerostomia/dry mouth-related protein–protein interactions, and (iv) determine drug–gene interactions potentially useful for xerostomia therapeutic development.

## 2. Methods

### 2.1. Conventional Review of the Literature

The PubMed and PubMed Central biomedical databases were used to search for articles in English relating to xerostomia and/or dry mouth [21]. We also sought to identify publications discussing the composition of saliva or providing information about what constitutes low-, optimal-, and high-quality saliva. From this review, we selected keywords, referred to as “search terms”, thought to be linked to xerostomia and/or saliva to a variable degree and risk of bias of each article was determined [22,23]. Search terms were used as input for the online tool pubmed2ensembl-biomartv0.7 (P2E) [24]. Results from P2E for each search term were then combined with P2E results for “xerostomia” or “dry mouth” and duplicates were removed.

### 2.2. Protein–Protein Interaction and Visualization

The Search Tool for Recurring Instances of Neighboring Genes (STRINGv11.0) was used to analyze protein–protein interaction (PPI) networks of the gene sets for xerostomia and dry mouth separately using the maximum confidence level (CL) of 0.90 [25]. All genes without interactions were removed from further analysis.

STRING PPI networks of candidate genes with a CL > 0.90 for xerostomia and dry mouth target lists were imported into Cytoscapev3.8.2 via the stringAppv1.6.0 [26,27]. Using the imported PPI networks from STRINGv11.0, Cytoscape PPI networks were constructed with a confidence score (CS) of 0.98 for xerostomia and dry mouth gene sets separately to narrow results. These networks were then merged to create a PPI intersection network of xerostomia and dry mouth genes with matching name or Ensembl ID. Functional and enrichment data for these networks were retrieved using Cytoscapev3.8.2:stringAppv1.6.0 [27]. The filtered enrichment categories chosen were Kyoto Encyclopedia of Genes and Genomes (KEGG) pathways and Gene Ontology (GO) Biological Process [28,29]. Using the BiNGOv3.0.3 Cytoscapev3.8.1app, a network of overrepresented GO biological processes was created for the xerostomia/dry mouth intersection network using a hypergeometric test and false discovery rate (*p* < 1.0 × 10^−4^) [30]. AmiGO 2 was used to explore overrepresented GO biological processes [31]. Tissue expression of genes found in the intersection network of xerostomia/dry mouth was retrieved and plotted using histograms via Pythonv3.6.1:matplotlibv3.0.3 [32,33]. Cytoscapev3.8.2:stringAppv1.6.0 extracts information from TISSUESv2.0 database, using a confidence rating (CR) from zero to five on 20 tissue types, based on knowledge, experiments and text mining [34].

### 2.3. The Drug–Gene Interaction Database

The drug–gene interaction database (DGIdb) was used to identify drug interactions for genes found in the xerostomia/dry mouth intersection network [34]. Drugs and genes identified by DGIdb were then compared to drugs that have been or are currently being evaluated for treatment of xerostomia or dry mouth according to the ClinicalTrials.gov database [35,36]. 

## 3. Results

**Figure 1:**
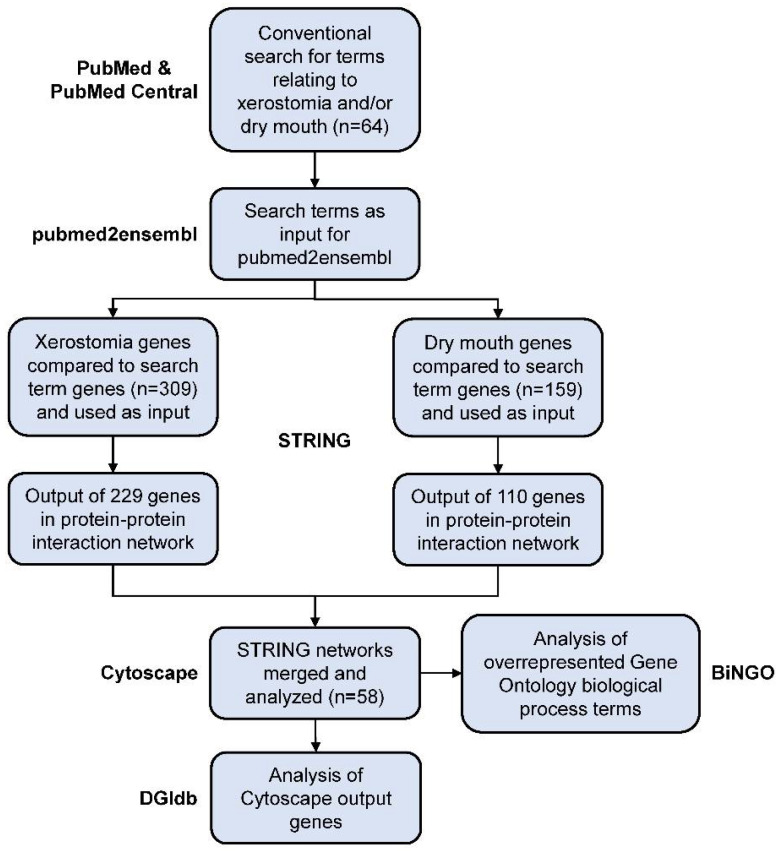
Overall Analytical Design. Search terms (*n* = 64) were determined to be related to xerostomia and/or dry mouth. Using search terms as input, pubmed2ensembl identified 1916 genes (309 without duplicates) and 1134 genes (159 without duplicates) to be related to xerostomia or dry mouth, respectively. Using STRINGv11.0, 229 genes were used as input for xerostomia and 110 genes were used as input for dry mouth with a confidence level of 0.90. STRINGv11.0 protein–protein interaction networks were used as input for Cytoscapev3.8.1 with a confidence score of 0.98. Network of 128 and 55 genes for xerostomia and dry mouth, respectively, were returned and merged, resulting in 58 genes with matching names or Ensembl IDs. The BiNGOv3.0.3 Cytoscapev3.8.1 app determined 360 gene ontology biological processes to be overrepresented in this intersection (*p* < 1.0 × 10^−4^). From 58 genes, 54 were found to have drug–gene interactions via the drug–gene interaction database (DGIdb). Using 54 total candidate gene targets, 27 drug trials consisting of 18 different drugs were identified using ClinicalTrials.gov that have been or are being evaluated for efficacy in treating xerostomia in general, head and neck cancer-related salivary gland dysfunction, or Sjögren’s Syndrome.

A flowchart showing the overall text- and data-mining methodology is presented in Figure 1. 

### 3.1. Identification of Genetic and Proteomic Profiles Predominantly Associated with Xerostomia/Dry Mouth

From conventional searches for xerostomia and dry mouth using PubMed and PMC, 64 search terms were determined as related (Table 1). A table showing risk of bias for reviewed and selected articles is presented in Table S1. Search terms (*n* = 64) were split into six categories directly related to the etiology and/or pathobiology of xerostomia and/or dry mouth (Table 1). These categories are (i) autoimmune disorders (*n* = 12 terms), (ii) diet (*n* = 10 terms), (iii) genetics and physiology (*n* = 17 terms), (iv) medication (*n* = 14 terms), (v) radiation (*n* = 3 terms), and (vi) others (*n* = 8 terms). P2E analysis returned 1916 gene symbols and aliases (309 without duplicates) for “xerostomia” and 1134 (159 without duplicates) for “dry mouth. P2E result outputs are presented in Data File S1.

### 3.2. Xerostomia/Dry Mouth Protein–Protein Interactions

**Figure 2:**
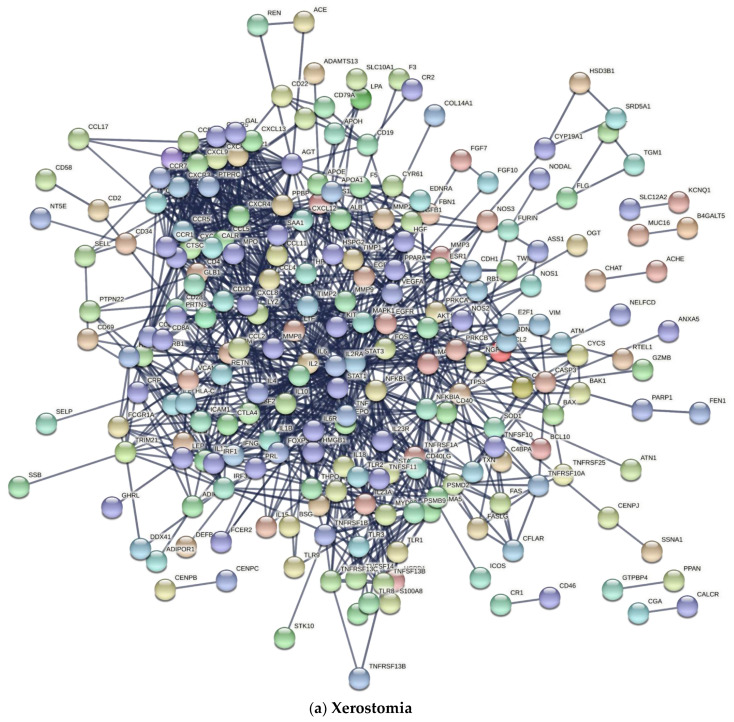

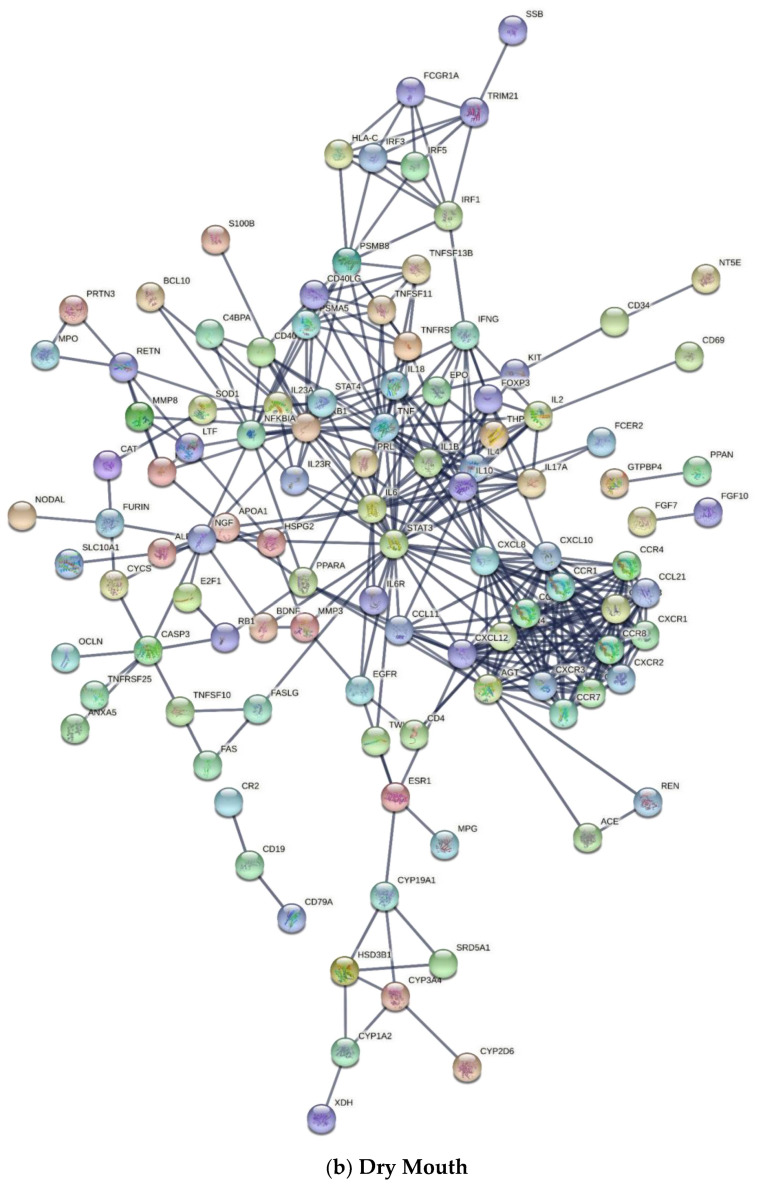
STRING PPI Networks for Xerostomia and Dry Mouth. STRINGv11.0 protein–protein interaction networks for genes identified using search terms combined with (**a**) xerostomia (input = 309 genes; output = 229) and (**b**) dry mouth (input = 159 genes; output = 110) at a confidence level of 0.90.

The STRINGv11.0 PPI network using a CL of 0.90 for the ‘xerostomia’ gene set (input = 309) returned a network of 229 genes (Figure 2a). PPI analysis using a CL of 0.90 for the ‘dry mouth’ gene set (input = 159) returned a network of 110 genes (Figure 2b).

**Figure 3:**
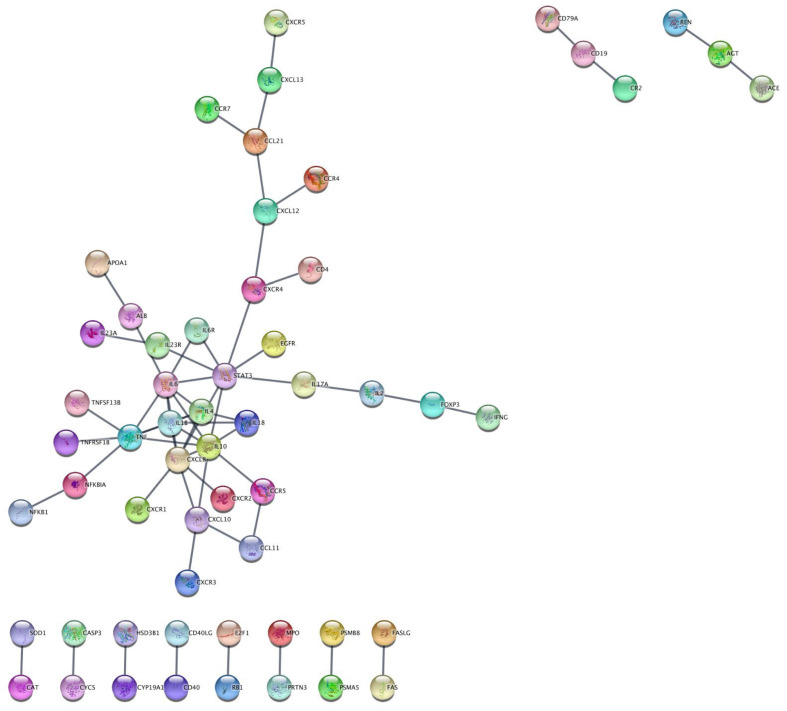
Cytoscape Protein–Protein Interaction Network of Xerostomia/Dry Mouth Network Intersection. Cytoscapev3.8.1:stringAppv1.6.0 protein–protein interaction network of xerostomia (input = 128 genes) and dry mouth (input = 55 genes) intersection (genes with matching name or Ensembl ID) with a 0.98 confidence score. The total number of returned genes in the network intersection is 58.

Using STRINGv11.0 PPI networks imported into Cytoscapev3.8.2:stringAppv1.6.0, PPI networks of 128 genes from 229 genes and 55 genes from 110 genes for xerostomia and dry mouth, respectively, were returned as output with a CS of 0.98 (Figure S1). Using the “merge networks” feature in Cytoscapev3.8.2, an intersection network was created of the xerostomia/dry mouth networks (Figure S1a,b), and a total of 58 genes were returned at a CS of 0.98 (Figure 3).

Of the 95 FDR significant (*p* < 1.0 × 10^−4^) KEGG pathways, cytokine–cytokine receptor interaction (hsa04060; FDR = 1.06 × 10^−49^), Hepatitis B (hsa05161; FDR = 1.89 × 10^−25^), and pathways in cancer (hsa05200; FDR = 1.29 × 10^−23^) were highly significant (Table S2). All significant KEGG pathways are presented in Data File S2. A total of 360 GO biological processes were determined as overrepresented, with the most significant being “immune system process” (GO:0002376; FDR = 2.61 × 10^−29^) and “inflammatory response” (GO:0006954; FDR = 4.16 × 10^−22^) (Data File S3). Although not significant, 31 GO biological processes were included in the overrepresented GO network due to being ‘parents’ of significant categories. A network of overrepresented GO biological processes is presented in Figure S2. 

Of the 20 available tissue types from the TISSUESv2.0 database, our dataset had confidence ratings for 19 tissue types [34]. Albumin (ALB) and apolipoprotein A1 (APO1) had the highest confidence rating of 5.00 in heart. ALB also had the highest confidence rating in liver. The highest gene expression confidence among all genes was determined to be from blood, with an average expression confidence rating of 4.09. Saliva had an average tissue expression confidence rating of 1.94 among all genes. In saliva, myeloperoxidase (MPO) had the highest saliva expression confidence rating of 4.35, with the second highest being a confidence rating of 2.80 for epidermal growth factor receptor (EGFR). Histograms highlighting gene expression confidence ratings for the 19 tissue types are presented in Figure S3.

### 3.3. The Drug–Gene Interaction Database

Using an input of 58 genes from the Cytoscape xerostomia/dry mouth intersection network, 54 genes were determined to have drug–gene interactions via DGIdb (Table 2) [34]. The gene with the greatest number of drug–gene interactions was EGFR, with 178 drug interactions (Table S3). The second greatest number of drug interactions was nuclear factor kappa B subunit 1 (NFKB1) with 90 interactions (Table S3). For the 54 total gene targets, 27 associated clinical trials consisting of 18 different drugs have been evaluated for treatment of xerostomia, head and neck cancer-related salivary gland dysfunction, or Sjögren’s Syndrome per the ClinicalTrials.gov database (Table S3) [36]. No clinical trials were found to evaluate drugs with EGFR as a target and only one clinical trial has evaluated a drug with NFKB1 as a target for Sjögren’s Syndrome (NCT00001599) [37]. Furthermore, of the 18 drugs in trials, 13 were also identified by DGIdb [35]. Only two of these 13 drugs identified by DGIdb had interaction scores greater than 10 [35]. These two drugs were belimumab (gene target tumor necrosis factor superfamily member 13b [TNFSF13B], with an interaction score of 63.79) and tocilizumab (gene target interleukin 6 receptor [IL6R], with an interaction score of 17.72) using the ratio of the average known gene partners for all drugs to the known partners for a given drug depending on publications. The highest interaction score among all identified DGIdb genes was for the drug mogamulizumab which targets the gene C–C Motif Chemokine Receptor 4 (CCR4) [35]. DGIdb identified 808 different drugs with gene interactions from our gene set that have not currently been evaluated for treatment of dry mouth, xerostomia, or Sjögren’s Syndrome [35]. Drugs identified by DGIdb and their interaction scores are available in Data File S4 [35].

## 4. Discussion

This is the first study to apply a conventional review with bioinformatics tools to determine genetic and proteomic panels regarding the treatment of xerostomia (Figure 1). This approach has the potential to identify drug targets which could be used to create targeted treatments for xerostomic patients. We were able to identify search terms related to xerostomia and/or dry mouth (Table 1) and to combine P2E results for either xerostomia or dry mouth, thereby producing large networks of PPIs using STRING_v11_._0_ at a high confidence (Figure 2). STRING_v11_._0_ produced PPI networks of 229 xerostomia and 110 dry mouth genes using a CL of 0.90. Using a CS of 0.98, Cytoscape_v3_._8_._1_ was able to narrow the STRING_v11_._0_ results by returning networks of 128 genes from 229 xerostomia genes and 55 genes from 110 dry mouth genes (Figure S1). Using the ‘intersection’ option in Cytoscape_v3_._8_._1_, we were able to focus our results to 58 genes from 128 xerostomia and 55 dry mouth genes with matching names or Ensembl IDs (Figure 3). Additionally, we were able to enrich our Cytoscape_v3_._8_._1_ results with KEGG pathways as well as GO biological processes and to determine possible drug targets from our results.

When investigating the significant KEGG pathways identified by Cytoscape, we found that the ‘cytokine–cytokine receptor interaction’ pathway (hsa04060) is related to ‘hypohidrotic ectodermal dysplasia’ (KEGG DISEASE: H00651) and ‘chronic mucocutaneous candidiasis’ (KEGG DISEASE: H01109), both conditions causing symptoms related to xerostomia (Table S2 and Data File S2) [28]. Furthermore, the ‘cytokine–cytokine receptor interaction’ pathway was the most significant KEGG pathway (hsa04060; FDR = 1.06 × 10^−49^) identified in the xerostomia/dry mouth intersection network. This pathway involves the gene *IL6R* which had the second-highest drug–gene interaction score of 17.72 (Table S2, Data File S2 and Data File S4) [35]. *IL6R* was also the target of a drug that we identified and has been evaluated in a clinical trial for its efficacy in primary Sjögren’s Syndrome (pSS) patients (NCT01782235) (Table 2) [38]. The clinical trial was completed in July of 2018 and although results have not been posted, a publication of the trial determined that tocilizumab did not improve symptoms over a 24 week period compared to the placebo [39]. Indeed, *IL6R* has eight other drug–gene interactions according to DGIdb, which might explain this outcome (Table S3) [35]. Additionally, *IL6R* is a gene product of the ‘inflammatory response’ biological process (GO:0006954) which was found to be the second most overrepresented using BiNGO (Data File S3) [29,30,31]. 

*IL6R* was also identified in the third-most significant KEGG pathway, ‘pathways in cancer’ (hsa05200) (Table S2) [28]. We found that this pathway also involves the genes *NFKB1* and *EGFR*. *NFKB1* had 90 drug interactions according to the DGIdb database (Table S3) [34]. Our analysis revealed that only one clinical trial had evaluated the drug thalidomide targeting *NFKB1* for Sjögren’s Syndrome (NCT00001599) (Table 2) [37]. This phase II trial was completed in June 2002, with no results posted. However, Pillemer et al. described that the administration of thalidomide was associated with unacceptable adverse effects when given at a dose of 50 or 100 mg [40]. All primary Sjögren’s Syndrome patients (*n* = 4) had to discontinue the medication after the third week [40]. Furthermore, no clinical trials were identified that have evaluated *EGFR* as a drug target in xerostomia, dry mouth, or Sjögren’s Syndrome. Indeed, *EGFR* had 178 drug–gene interactions in our gene set (the maximum), which could be exploited to partially restore the functionality of salivary glands by reducing inflammation (Table 2 and Table S3). We determined that *EGFR*, a gene belonging to the ‘inflammatory response’ biological process (GO:0006954), had the second-highest salivary expression confidence rating of 2.80 (Figure S3 and Data File S3) [29,31]. In addition, Lisi et al. determined that overexpression of nerve growth factor-beta, which is elevated in salivary gland epithelial cells in pSS, can be prevented with *EGFR* pathway inhibition [41]. Furthermore, Sisto et al. found that pro-inflammatory cytokine release can be reduced in salivary gland epithelial cells using an *EGFR* inhibitor combined with an *ADAM metallopeptidase domain 17* (*ADAM17*) inhibitor [42]. Further investigation into the involvement of these pathways and genes in xerostomia are warranted.

Other genes that may be of interest in the development and treatment of xerostomia are *MPO* and *TNFSF13B*, both of which are involved in the significantly overrepresented GO ‘immune system process’ (GO:002376) (Data File S3) [29,31]. Although *MPO* was not identified in a highly significant KEGG pathway, it had the highest expression confidence rating of 4.35 in saliva and had 21 drug–gene interactions via DGIdb (Figure S3 and Table S3) [35]. Moreover, *MPO* was a target of drugs in five clinical trials evaluating their effectiveness in the treatment of xerostomia, head and neck cancer oral complications, and Sjögren’s Syndrome (NCT02430298, NCT00057785, NCT03953703, NCT04392622, and NCT02990468) (Table 2) [43,44,45,46,47]. One clinical trial determined that intensity modulated radiation therapy (IMRT) in patients with nasopharyngeal cancer combined with cisplatin and fluorouracil improved xerostomia scores significantly compared to previous radiation therapy oncology group trials [48]. Although some studies suggest that melatonin may have implications in the prevention and/or treatment of oral pathologies such as xerostomia, no clinical trials have reported its efficacy [49,50,51]. Clinical trials involving cisplatin, melatonin, and/or levocarnitine still hold the promise for better outcomes in the prevention and treatment of xerostomia. In addition, for disease conditions such as diabetes mellitus causing salivary gland dysfunction which results in salivary flow reduction and a change in saliva composition, significant mitigation of subjective xerostomia may be achieved through artificial saliva [52].

*TNFSF13B* is another gene of interest related to xerostomia, as, in our analysis, it had the greatest drug–gene interaction score of 63.79 among drugs that have been/are being evaluated in clinical trials (Data File S4). It is also the only identified target of the drug belimumab (Data File S4) [35,53]. Two clinical trials were found that evaluated this drug for efficacy and safety in pSS patients (NCT02631538 and NCT01160666) (Table 2) [54,55]. In clinical trial NCT02631538, of 24 patients receiving belimumab monotherapy, two patients had adverse effects and only one patient reached the stopping criteria for the study [54]. The other clinical trial (NCT01160666) found that lower blood cells and salivary natural killer cells were associated with a better response to belimumab [55,56]. This could be due to the high expression confidence of *TNFSF13B* in blood and saliva, 4.63 and 2.54, respectively (Figure S3). Furthermore, in patients with kidney renal clear cell carcinoma, it has been suggested that *TNFSF13B* may regulate the natural kill cell-mediated cytotoxicity pathway [57]. 

## 5. Limitations

For this study, several limitations related to our methodology and results must be recognized. There are few publications on xerostomia and limited human trials investigating the condition. Furthermore, xerostomia pathobiology is not well understood and there is much debate among investigators regarding the prevalence of xerostomia in different geographical locations and ethnic groups. The results reported in this study that may be relevant to xerostomia therapeutic development will require substantial validation in experimental preclinical models, since these were based on ‘confidence’ ranking of published research and scores provided by computational systems biology tools.

## 6. Conclusions

The *IL6R*, *EGFR*, *NFKB1*, *MPO*, and *TNFSF13B* genes might all have implications in the diagnosis and intervention of xerostomia. Our findings highlight the need for further investigation into these genes as candidate targets for treatment or treatment response follow-up in the future. However, the greatest challenge of such investigations resides in the difficulty to screen for relevant biomarkers, including those associated with genetic susceptibility, which would enable a better stratification of xerostomic patients at the molecular level.

## Figures and Tables

**Table 1:** Xerostomia and/or Dry Mouth Search Terms. Search terms (*n* = 64) were determined using conventional methods. PubMed and PubMed Central were used to search for articles in English relating to xerostomia and/or dry mouth. Keywords or “search terms” thought to be linked to xerostomia and/or saliva were selected. Search terms were split into categories directly related to the etiology and/or pathobiology of xerostomia and/or dry mouth: ^a^ autoimmune conditions; ^b^ dietary and nutritional; ^c^ genetic and physical; ^d^ medication; ^e^ radiation therapy applied to head and neck cancer (HNC) patients; ^f^ other.

Category	Search Term (*n* = 64)
Autoimmune Disease ^a^	Amyloid build up, chronic inflammatory autoimmune disorder, chronic juvenile arthritis, crest syndrome, dry mouth, hypohidrotic ectodermal dysplasia, juvenile rheumatoid arthritis, sarcoidosis, scleroderma, Sjögren’s Syndrome; underactive thyroid gland, xerostomia (*n* = 12)
Diet ^b^	Alcohol use, caffeine drink, food avoidance, food modification, morbidly obese, nutritional benefit, specialized metabolites, sugared beverages, tobacco use, unhealthy eating (*n* = 10)
Genetics and Physiology ^c^	Burning mouth, difficulty swallowing, glycosylation, high flow salivary hypofunction, high-quality saliva, low-quality saliva, normal salivary composition, oncogenomics, optimal salivary function, poor saliva composition, saliva composition, salivary flow pH 5.5, salivary flow pH 7.8, salivary device, salivary flow between 0.4 mL and 0.5 mL, salivary hypofunction, sore throat (*n* = 17)
Medication ^d^	Acetylcholine blocker, amphetamines, atropine, biotene, gustatory stimulants, histamine receptor inhibitor, lozenge, mucosal coating agent, opioid drug class, parasympathomimetic prescription, replacement saliva, saliva substitute, serotonin reuptake inhibition, valium (*n* = 14)
Radiation ^e^	Radiation therapy, RTx, XRT (*n* = 3)
Other ^f^	Feverishness, free water loss, impoverishment, multicultural populations, oral health care, oral thrush, thallium poisoning, vitamin A deficiency (*n* = 8)

**Table 2:** Drugs Evaluated via ClinicalTrials.gov and the Drug–Gene Interaction Database Identified Drug–Gene Interactions. ^a^ Drug identified by Clinicaltrials.gov (https://ClinicalTrials.gov/) that has been/is being evaluated for diseases related to xerostomia/dry mouth. ^b^ Disease that the drug has been/is being evaluated for with an input search in ClinicalTrials.gov of “xerostomia” and “dry mouth”. ^c^ ClinicalTrials.gov identifier. ^d^ Gene targets of drug identified using DrugBank (https://go.drugbank.com). Genes shown in bold are in common with the 54 genes identified with interactions using the Drug–Gene Interaction database (DGIdb) (https://www.dgidb.org/). ^e^ Genes identified by DGIdb (https://www.dgidb.org/) as having interactions with the drug. Note: HNC-OC is head and neck cancer oral complications; ND is not determined; SS is Sjögren’s Syndrome.

Drug ^a^	Disease ^b^	Clinical Trial ID ^c^	DrugBank Identified Drug Target ^d^	DGIdb Gene ^e^
Belimumab	SS	NCT02631538; NCT01160666	*TNFSF13B*	*TNFSF13B*
Cisplatin	HNC-OC; Xerostomia	NCT00057785; NCT04392622; NCT02990468	*DNA; MPG; A2M; TF; ATOX1; **MPO**; XDH; CYP4A11; PTGS2; NAT; CYP2C9; CYP2B6; BCHE; GSTT1; MT1A; MT2A; **SOD1**; GSTP1; NQO1; GSTM1; **ALB**; ABCC3; ABCC5; ABCC2; SLC22A2; SLC31A1; SLC31A2; ABCC6; ATP7B; ATP7A; ABCG2*	*E2F1; CXCR4; IL6; RB1; IFNG; EGFR; FAS*
Cyclosporine	SS	NCT00025818	*HRH1; HRH2; HRH3; S100A1; S100A2; S100B; S100A13; S100A2; CYP3A4; FMO1; FMO3; **ALB**; ABCB1*	*IL2; TNF; IL10*
Dexamethasone	SS; Xerostomia; HNC-OC	NCT01316770; NCT01748942; NCT00631358	*NR3C1; NR0B1; ANXA1; NOS2; NR1I2; HSD11B2; CYP3A4; HSD11B1; CYP3A5; CYP3A7; CYP17A1; CYP1A1; CYP2A6; CYP2B6; CYP2C19; CYP2C8; CYP2E1; CYP3A43; CYP4A11; CYP11B1; **ALB**; ABCB1; SLC22A8; ABCB11; ABCC2; ABCG2; SLCO1A2*	ND
Etanercept	SS	NCT00001954	***TNF**; FCGR1A; FCGR2A; FCGR2B; FCGR2C; FCGR3A; FCGR3B; LTA; C1Q PROTEIN GROUP*	*TNFRSF1B*
Fluorouracil	HNC-OC	NCT00057785	*TYMS; DNA; RNA; CYP2C9; CYP1A2; TYMP; DPYD; UPP1; UPP2; CYP2A6; CYP2C8; MTHFR; TYMS; UMPS; PPAT; **ALB**; SERPINA7; SLC22A7; SLC29A1; ABCG2; ABCC3; ABCC4; ABCC5*	*IL6R*
Hydroxychloroquine	SS	NCT01601028; NCT00431041; NCT00873496	*DNA; TLR7; TLR9; ACE2; CYP3A4; CYP2D6; CYP2C8; **ALB***	*TNF*
Levocarnitine	SS	NCT03953703	*SLC22A4; SLC22A5; CRAT; SLC24A29; SLC25A20; CROT; CPT2; CPT1A; XDH; CES1; **MPO**; SLC22A4; SLCO1B1; SLC22A5; SLC22A16; SLC22A8*	ND
Melatonin	HNC-OC	NCT02430298	*MTNR1A; MTNR1B; ESR1; RORB; CALM1; **MPO**; EPX; CALR; ASMT; NQO2; CYP1A1; CYP1A2; CYP1B1; CYP2C19; CYP2C9; ASMT; IDO1; **CYP19A1**; SLC22A8*	*IFNG*
Methotrexate	Autoimmune diseases	NCT03239600	*DHFR; TYMS; ATIC; DHFR; AOX1; MTHFR; PGD; FPGS; TYMS; ATIC; GGH; CYP3A4; **ALB**; ABCC3; ABCC4; ABCC1; SLC22A6; ABCC10; SLC22A8; ABCC2; ABCB1; SLC01A2; SLC16A1; ABCC11; SLCO1B3; SLC22A11*; *SLCO1C1*; *SLCO3A1*; *ABCG2*; *SLC22A7*; *SLC46A1*; *SLCO1B1*; *SLC04C1*; *SLC19A1*; *FOLR1*; *FOLR2*; *SLC15A1*; *SLC36A1*	*FOXP3*; *RB1*; *IL2*; *E2F1*
Mirabegron	SS	NCT04909255	*ADRB3*; *ADRB1*; *CYP3A4*; *CYP2D6*; *BCHE*; *UGT2B7*; *UGT1A3*; *UGT1A8*; ***ALB***; *ORM1*; *ABCB1*; *SLCO1A2*; *SLC22A1*; *SLC22A2*; *SLC22A3*	ND
Oxybutynin	SS; Overactive bladder	NCT04909255; NCT00431041	*CHRM3*; *CHRM2*; *CHRM1*; *CYP3A4*; *CYP2C8*; *CYP2D6*; *CYP3A5*; *ORM1*; ***ALB***	ND
Paclitaxel	Xerostomia	NCT00095927	*TUBB1*; *BCL2*; *MAP4*; *MAP2*; *MAPT*; *NR1I2*; *CYP3A4*; *CYP3A5*; *CYP3A7*; ***CYP19A1***; *CYP1B1*; *CYP2C8*; *ABCB11*; *ABCB1*; *ABCC1*; *ABCC10*; *SLCO1B3*; *ABCC2*	*E2F1*; *CYP19A1*; *CASP3*; *EGFR*; *CXCL8*
Prednisone	SS	NCT02370550	*NR3C1*; *CYP3A4*; *CYP2C19*; *CYP3A5*; *CYTOCHROME P450 3A SUBFAMILY GROUP*; *CYP2A6*; *CYP1B1*; *CYP2B6*; *CYP2CB*; *CYP2C9*; *HSD11B1*; ***ALB***; *SERPINA6*	*CXCL12*; *IFNG*
Tacrolimus	SS; Dry eye	NCT03865888; NCT01850979	*FKBP1A*; *CYP3A5*; *CYP3A4*; ***ALB***; *ORM1*; *ABCB1*; *ABCA5*; *SLCO1B1*	*IL18*; *FOXP3*; *IL10*
Thalidomide	SS; Xerostomia	NCT00001599	*CRBN*; ***TNF***; ***NFKB1***; *DNA*; *FGFR2*; *PTGS2*; *ALPHA1-ACID GLYCOPROTEIN GROUP*	*IL6R*; *NFKB1*; *TNF*
Tocilizumab	SS	NCT01782235	***IL6R***; *CYP3A4*	*IL6R*
Tofacitinib	SS	NCT04496960	*JAK1*; *JAK2*; *JAK3*; *TYK2*; *CYP3A4*; *CYP2C19*; ***ALB***	ND

## Data Availability

Publicly available data were used. All data used can be retrieved from the supplementary files or through our lab’s GitHub repository (https://github.com/mbeckm01/Xerostomia.git, accessed on 12 January 2022).

## Supplementary Materials

The following supporting information can be downloaded at: https://www.mdpi.com/article/10.3390/jcm11051442/s1, Table S1: Risk-of-Bias Assessment per PRISMA Guidelines. Risk-of-Bias assessment table using ‘Cochran’s Handbook for Systematic Reviews of Interventions’ Risk-of-bias VISualization (robvis) tool for 40 publications manually curated using conventional searches of PubMed and PubMed Central for articles related to xerostomia, in English, between the years 2000 and 2020. Using Cochran’s Risk-of-Bias 2 criteria, 19 publications were excluded. Of the total 40 publications, 21 publications were investigated to determine ‘search terms’ for xerostomia and dry mouth. Data File S1: P2E Results for Search Terms. Pubmed2ensembl results for 64 identified search terms. Figure S1: Cytoscape Xerostomia and Dry Mouth Protein–Protein Interaction Networks. Cytoscape_v3_._8_._1_ protein–protein interaction networks using stringApp_v1_._6_._0_ for (a) xerostomia (input = 229 genes; output = 128 genes) and (b) dry mouth (input = 110 genes; output = 55 genes) with a confidence score of 0.98 and a tissue expression filter for saliva set at 1.0. Data File S2: KEGG Pathways via STRING Enrichment from Cytosape for Xerostomia/Dry Mouth Intersection. Data File S3: GO Overrepresented Biological Processes from Cytoscape for Xerostomia/Dry Mouth Intersection. Table S2: Significant KEGG Pathways via STRING Enrichment in Cytoscape for Xerostomia/Dry Mouth Intersection. Table showing FDR significant (*p* < 1.0 × 10^−4^) KEGG pathways with more than 25 genes via Cytoscape_v3_._8_._1_:stringApp_v1_._6_._0_ STRING enrichment for xerostomia/dry mouth interaction network at a confidence score of 0.98. Genes in bold are in the gene xerostomia/dry mouth interaction gene set. Figure S2: BiNGO Overrepresentation of GO Terms for Xerostomia/Dry Mouth Intersection. Biological Networks Gene Ontology (BiNGO_v3_._0_._3_) tool network of overrepresented Gene Ontology (GO) terms (*n* = 360) in the xerostomia/dry mouth intersection protein–protein interaction network for humans. Overrepresented GO terms shown are those at *p* < 1 × 10^−4^ using a hypergeometric test and false discovery rate correction. Figure S3: Gene Expression Confidences for 19 out of 20 Tissue Types. Histograms plotted using Python_v3_._6_._1_:matplotlib_v3_._0_._3_ package showing Cytoscape_v3_._8_._2_ stringApp_v1_._6_._0_ network gene expression for 19 out of 20 possible tissue types identified in the PPI network of xerostomia/dry mouth intersection with a confidence score of 0.98. Gene expression data from stringApp_v1_._6_._0_ STRING enrichment feature that gathers information about expression of genes from TISSUES_v2_._0_ database (https://tissues.jensenlab.org/Search, accessed on 8 August 2021). TISSUES_v2_._0_ uses a “confidence” based rating from zero to five based on knowledge, experiments, and text mining. Table S3: The Drug–Gene Interaction Database Identified Drug–Gene Interaction Counts. ^a^ Gene identified in the xerostomia/dry mouth intersection network (54 of 58). ^b^ Number of drug–gene interactions identified using the Drug–gene Interaction database. Data File S4: The Drug–Gene Interaction Database Identified Interactions.
