# Supplementary material for: A Computational Text Mining-Guided Meta-Analysis Approach to Identify Potential Xerostomia Drug Targets
Source: J Clin Med. 2022 Mar 5;11(5):1442. doi: 10.3390/jcm11051442 (PMC8911392; doi:10.3390/jcm11051442)
Supplement: Supplementary file 1 [file jcm-11-01442-s001.zip › Figure S1. Cytoscape Xerostomia Dry mouth MB 06 09 21.pdf]

Figure S1. Cytoscape Xerostomia and Dry Mouth protein-protein interaction networks

a. Xerostomia Cytoscape stringApp network

b. Dry Mouth Cytoscape stringApp network

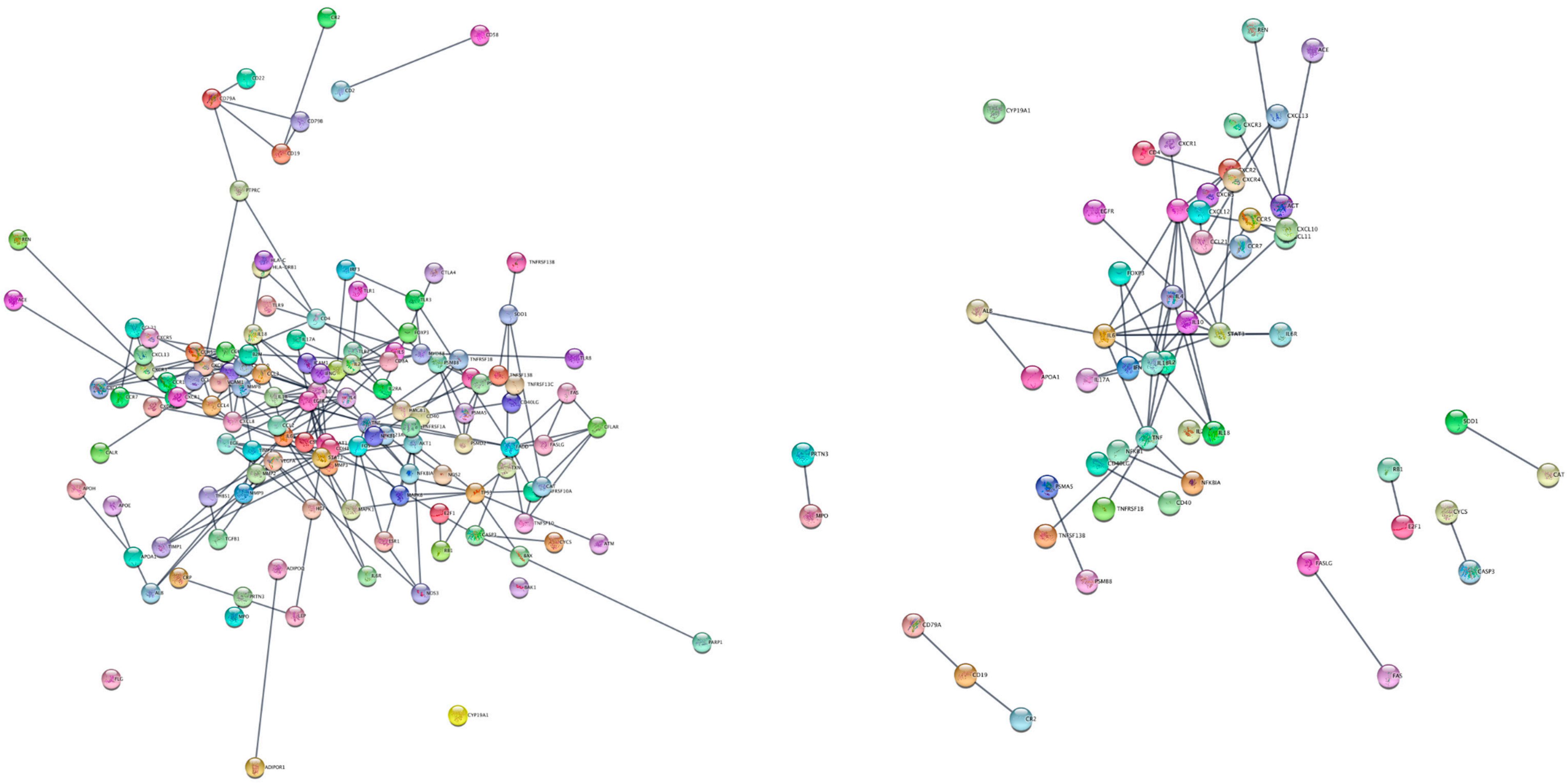

Legend. Cytoscape$_{v3.8.1}$ protein-protein interaction networks using stringApp$_{v1.6.0}$ for **a.** Xerostomia and **b.** Dry mouth with a confidence of 0.98 and a tissue expression filter for saliva set at 1.0.
