# Supplementary material for: A Computational Text Mining-Guided Meta-Analysis Approach to Identify Potential Xerostomia Drug Targets
Source: J Clin Med. 2022 Mar 5;11(5):1442. doi: 10.3390/jcm11051442 (PMC8911392; doi:10.3390/jcm11051442)
Supplement: Supplementary file 1 [file jcm-11-01442-s001.zip › Figure S2. BiNGO Intersection Network MB 06 09 21.pdf]

Figure S2. BiNGO overrepresentation of GO terms for Xerostomia/Dry Mouth Intersection**.**

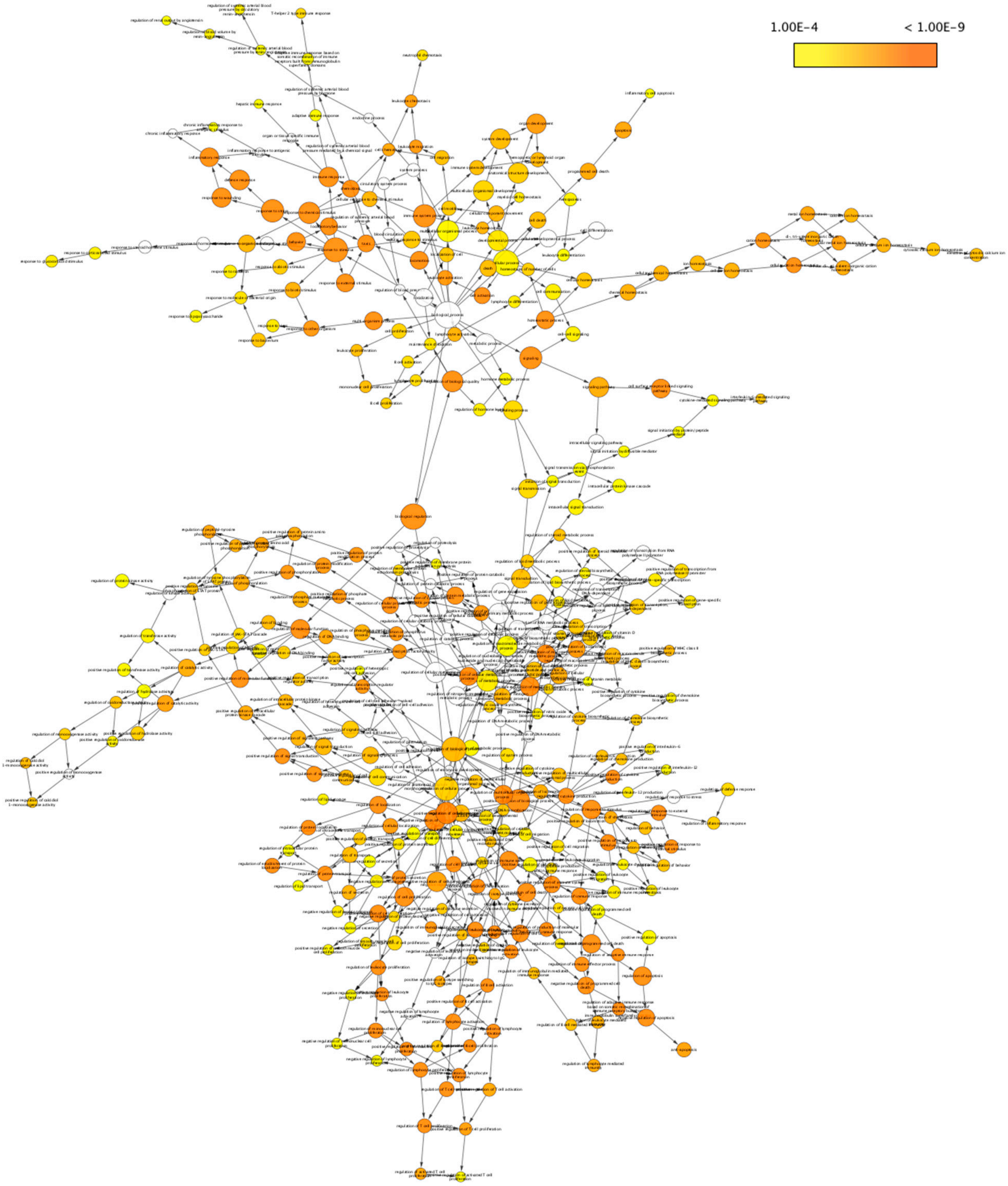

Legend. Biological Networks Gene Ontology (BiNGO$_{v3.0.3}$) tool network of overrepresented Gene Ontology (GO) terms (n=360) in the xerostomia/dry mouth intersection protein-protein interaction network for humans. Overrepresented GO terms shown are those at $p<1x10^{-4}$ using a hypergeometric test and false discovery rate correction.
