# Supplementary material for: A Computational Text Mining-Guided Meta-Analysis Approach to Identify Potential Xerostomia Drug Targets
Source: J Clin Med. 2022 Mar 5;11(5):1442. doi: 10.3390/jcm11051442 (PMC8911392; doi:10.3390/jcm11051442)
Supplement: Supplementary file 1 [file jcm-11-01442-s001.zip › Table S1. Xerostomia Risk of Bias 2 MB 06 17 21.pdf]

Table S1. Risk of bias assessment per PRISMA guidelines

Study	D1	D2	D3	D4	D5	Overall
Field et al., 2001	-	?	+	+	+	+
Shinkai et al., 2006	-	?	+	+	+	+
Orellana et al., 2006	+	+	+	+	+	+
Dirix et al., 2008	X	+	+	+	+	+
Mirzaii-Dizgah et al., 2011	-	+	-	+	+	+
Furness et al., 2011	+	+	-	-	+	+
Alevizos et al., 2011	X	+	+	+	+	+
Sasportas et al., 2013	+	+	-	-	+	+
Pinna et al., 2015	+	+	-	+	+	+
Lopez-Pintor et al., 2016	+	+	-	+	+	+
Shah et al., 2017	-	+	+	+	+	+
Niklander et al., 2017	-	+	+	-	+	+
Mercadante et al., 2017	+	+	+	+	+	+
Park et al., 2018	+	+	-	+	+	+
Assery, 2019	+	+	-	+	+	+
Chen et al., 2019	X	+	+	+	+	+
Ni et al., 2019	+	+	-	+	+	+
Nascimento et al., 2019	-	+	+	-	+	+
Teng et al., 2019	-	+	+	+	+	+
Marcott et al., 2020	-	+	-	+	+	+
Wang et al., 2020	-	+	-	-	+	+

Risk of bias domains

Domains:
D1: Bias arising from the randomization process.
D2: Bias due to deviations from intended intervention.
D3: Bias due to missing outcome data.
D4: Bias in measurement of the outcome.
D5: Bias in selection of the reported result.

Judgement
X High
- Some concerns
+ Low
? No information

Footnote:

Risk-of-Bias assessment table using ‘Cochran’s Handbook for Systematic Reviews of Interventions’ Risk-of-bias VISualization (robvis) tool for 40 publications manually curated using conventional searches of PubMed and PubMed Central for articles related to xerostomia, in English, between the years 2000 and 2020. Using Cochran’s Risk-of-Bias 2 criteria, 19 publications were excluded. Of the total 40 publications, 21 publications were investigated to determine ‘search terms’ for xerostomia and dry mouth.
