# Supplementary material for: A Computational Text Mining-Guided Meta-Analysis Approach to Identify Potential Xerostomia Drug Targets
Source: J Clin Med. 2022 Mar 5;11(5):1442. doi: 10.3390/jcm11051442 (PMC8911392; doi:10.3390/jcm11051442)
Supplement: Supplementary file 1 [file jcm-11-01442-s001.zip › Table S2. KEGG pathways MB 06 11 21.pdf]

Table S2. Significant KEGG pathways via STRING enrichment in Cytoscape for xerostomia/dry mouth intersection

KEGG pathway name	KEGG ID	FDR adjusted p-value	Genes
Cytokine-cytokine receptor interaction	hsa04060	1.06×10^{-49}	*TNFRSF1A; THPO; CCL17; TNFRSF10A; TGFB1; HGF; CCL2; **IL2**; IL23A; **IFNG**; **IL4**; IL5; TNFSF10; **CCR7**; EPO; **CCL21**; TNFRSF13B; **IL1B**; EGF; **EGFR**; IFNA1; **IL18**; **CXCL13**; KIT; TNFRSF13C; **CXCR5**; **CCR5**; **CXCR1**; PPBP; CCR1; IL15; CSF2; PRL; **CCL11**; **CXCL10**; **CXCL8**; LEP; **CXCR2**; **IL23R**; CCR8; **CCR4**; **IL17A**; **FAS**; CXCL9; **FASLG**; **IL6R**; **CD40LG**; **CD40**; **CXCR3**; **TNFSF13B**; **TNFRSF1B**; TNFRSF25; IL2RA; **CXCL12**; TNFSF11; **IL6**; **CXCR4**; **TNF**; **IL10**; TNFSF14; CCL5; CCL3; VEGFA; CCL4*
Hepatitis B	hsa05161	1.89×10^{-25}	*MAPK1; **NFKBIA**; TGFB1; **NFKB1**; TLR2; **STAT3**; **RB1**; TP53; IFNA1; BAX; TLR3; FADD; PRKCB; FOS; **CXCL8**; **CYCS**; **CASP3**; **E2F1**; **FAS**; STAT1; **FASLG**; MMP9; TLR4; HSPG2; STAT4; MAPK8; BCL2; **IL6**; **TNF**; MYD88; PRKCA; AKT1; IRF3; CREB3L1*
Pathways in cancer	hsa05200	1.29×10^{-23}	*MAPK1; **NFKBIA**; MMP2; TGFB1; HGF; **NFKB1**; **IL2**; IL23A; **IFNG**; **IL4**; IL5; EPO; CDH1; **STAT3**; FGF10; EGF; **RB1**; FGF7; TP53; **EGFR**; IFNA1; KIT; BAX; IL15; FADD; PRKCB; FOS; **CXCL8**; **CYCS**; **CASP3**; EDNRA; **IL23R**; NOS2; **E2F1**; **FAS**; STAT1; TPR; **FASLG**; **IL6R**; MMP9; BAK1; IL2RA; STAT4; MAPK8; **CXCL12**; BCL2; IL6; **CXCR4**; ESR1; PRKCA; AKT1; VEGFA*
Chagas disease	hsa05142	1.29×10^{-23}	*TNFRSF1A; MAPK1; **NFKBIA**; TGFB1; CCL2; **NFKB1**; **IL2**; **IFNG**; TLR2; **IL1B; ACE**; CD3D; FADD; FOS; **CXCL8**; CFLAR; CALR; NOS2; **FAS**; **FASLG**; TLR4; MAPK8; **IL6**; **TNF**; MYD88; **IL10**; AKT1; CCL5; CCL3*
Rheumatoid arthritis	hsa05323	5.30×10^{-22}	*TGFB1; CCL2; IL23A; **IFNG**; TLR2; **IL1B**; ICAM1; **IL18**; IL15; CSF2; MMP3; CTLA4; FOS; **CXCL8**; CD28; **IL17A**; HLA-DRB1; TLR4; **TNFSF13B**; **CXCL12**; TNFSF11; **IL6**; **TNF**; CCL5; CCL3; VEGFA*
Toll-like receptor signaling pathway	hsa04620	1.96×10^{-21}	*MAPK1; **NFKBIA**; **NFKB1**; TLR2; **IL1B**; IFNA1; TLR3; FADD; **CXCL10**; FOS; **CXCL8**; TLR8; IRF5; STAT1; CXCL9; TLR1; **CD40**; TLR4; MAPK8; **IL6**; **TNF**; MYD88; AKT1; IRF3; CCL5; CCL3; CCL4*
Kaposi's sarcoma-associated herpesvirus infection	hsa05167	9.37×10^{-21}	*TNFRSF1A; MAPK1; **NFKBIA**; **NFKB1**; **STAT3**; ICAM1; **RB1**; TP53; IFNA1; **CCR5**; BAX; CCR1; TLR3; CSF2; FADD; FOS; **CXCL8**; **CYCS**; **CASP3**; CCR8; **CCR4**; **E2F1**; **FAS**; STAT1; BAK1; HLA-C; MAPK8; **IL6**; MICA; AKT1; IRF3; VEGFA*
Influenza A	hsa05164	9.37×10^{-21}	*TNFRSF1A; MAPK1; **NFKBIA**; TNFRSF10A; CCL2; **NFKB1**; **IFNG**; TNFSF10; **IL1B**; ICAM1; IFNA1; **IL18**; TLR3; PRKCB; **CXCL10**; **CXCL8**; **CYCS**; **FAS**; HLA-DRB1; STAT1; **FASLG**; TLR4; MAPK8; IL6; **TNF**; MYD88; PRKCA; AKT1; IRF3; CCL5; FURIN*
Tuberculosis	hsa05152	1.54×10^{-20}	*TNFRSF1A; MAPK1; TGFB1; **NFKB1**; IL23A; **IFNG**; TLR2; **IL1B**; IFNA1; **IL18**; BAX; FADD; **CYCS**; **CASP3**; NOS2; HLA-DRB1; STAT1; TLR1; CR1; FCGR1A; BCL10; TLR4; HSPD1; MAPK8; BCL2; **IL6**; **TNF**; MYD88; **IL10**; VDR; AKT1*
Chemokine signaling pathway	hsa04062	3.89×10^{-19}	*MAPK1; **NFKBIA**; CCL17; CCL2; NFKB1; **CCR7**; **CCL21**; **STAT3**; **CXCL13**; **CXCR5**; **CCR5**; **CXCR1**; PPBP; CCR1; **CCL11**; PRKCB; **CXCL10**; **CXCL8**; **CXCR2**; CCR8; **CCR4**; STAT1; CXCL9; **CXCR3**; **CXCL12**; **CXCR4**; AKT1; CCL5; CCL3; CCL4*
Apoptosis	hsa04210	4.55×10^{-18}	*TNFRSF1A; MAPK1; GZMB; **NFKBIA**; TNFRSF10A; **NFKB1**; CTSC; TNFSF10; TP53; ATM; BAX; FADD; FOS; **CYCS**; **CASP3**; CFLAR; **FAS**; PARP1; **FASLG**; NGF; BAK1; MAPK8; BCL2; **TNF**; DDIT3; AKT1*
Herpes simplex infection	hsa05168	2.42×10^{-16}	*TNFRSF1A; **NFKBIA**; CCL2; **NFKB1**; **IFNG**; TLR2; **IL1B**; TP53; IFNA1; IL15; TLR3; FADD; FOS; **CYCS**; **CASP3**; **FAS**; HLA-DRB1; STAT1; **FASLG**; HLA-C; MAPK8; **IL6**; **TNF**; MYD88; TNFSF14; IRF3; CCL5*
PI3K-Akt signaling pathway	hsa04151	1.80×10^{-12}	*MAPK1; HGF; **NFKB1**; **IL2**; **IL4**; EPO; TLR2; THBS1; FGF10; EGF; FGF7; TP53; **EGFR**; IFNA1; KIT; NOS3; PRL; **FASLG**; **IL6R**; NGF; TLR4; IL2RA; BCL2; IL6; PRKCA; BDNF; **CD19**; AKT1; VEGFA; CREB3L1*
MAPK signaling pathway	hsa04010	6.06×10^{-12}	*TNFRSF1A; MAPK1; TGFB1; HGF; **NFKB1**; **IL1B**; FGF10; EGF; FGF7; TP53; **EGFR**; KIT; PRKCB; FOS; **CASP3**; **FAS**; **FASLG**; NGF; MAPK8; **TNF**; MYD88; PRKCA; BDNF; FLNB; DDIT3; AKT1; VEGFA*
Human papillomavirus infection	hsa05165	1.45×10^{-10}	*TNFRSF1A; MAPK1; NFKB1; IRF1; THBS1; EGF; **RB1**; TP53; **EGFR**; IFNA1; ATM; BAX; TLR3; FADD; **CASP3**; **E2F1**; **FAS**; STAT1; **FASLG**; BAK1; HLA-C; **TNF**; AKT1; IRF3; VEGFA; CREB3L1*

Footnote. Table showing FDR significant ($p<1.0 \times 10^{-4}$) KEGG pathways with more than 25 genes via Cytoscape$_{v3.8.1}$:stringApp$_{v1.6.0}$ STRING enrichment for xerostomia/dry mouth interaction network. Genes in bold are in the gene xerostomia/dry mouth gene set.
