# Supplementary material for: A Computational Text Mining-Guided Meta-Analysis Approach to Identify Potential Xerostomia Drug Targets
Source: J Clin Med. 2022 Mar 5;11(5):1442. doi: 10.3390/jcm11051442 (PMC8911392; doi:10.3390/jcm11051442)
Supplement: Supplementary file 1 [file jcm-11-01442-s001.zip › Table S3. DGIdb counts of drug gene interactions MB 11 17 21.pdf]

Table S3. Drug-Gene Interaction database identified drug-gene interaction counts

Gene[a]	# of drug-gene interactions[b]
ACE	66
AGT	14
ALB	21
APOA1	8
CASP3	33
CAT	1
CCL11	3
CCL21	1
CCR4	3
CCR5	15
CD19	13
CD4	9
CD40	11
CD40LG	11
CR2	1
CXCL10	12
CXCL12	7
CXCL13	1
CXCL8	56
CXCR1	5
CXCR2	11
CXCR3	1
CXCR4	20
CYP19A1	36
E2F1	16
EGFR	178
FAS	19
FASLG	1
FOXP3	3
HSD3B1	1
IFNG	17
IL10	17
IL17A	16
IL18	7
IL1B	37
IL2	32
IL23A	6
IL23R	1
IL4	4
IL6	25
IL6R	9
MPO	21
NFKB1	90
NFKBIA	9
PRTN3	2
PSMA5	5
PSMB8	8
RB1	23
REN	8
SOD1	4
STAT3	23
TNF	68
TNFRSF1B	11

Footnote. [a]Drugs identified in the xerostomia/dry mouth intersection network. [b]Number of drug-gene interactions identified using the Drug-Gene Interaction database
