# Supplementary figures and images for: A Computational Text Mining-Guided Meta-Analysis Approach to Identify Potential Xerostomia Drug Targets
Source: J Clin Med. 2022 Mar 5;11(5):1442. doi: 10.3390/jcm11051442 (PMC8911392; doi:10.3390/jcm11051442)

**Figure S3.** Gene Expression confidences for 19 out of 20 tissue types

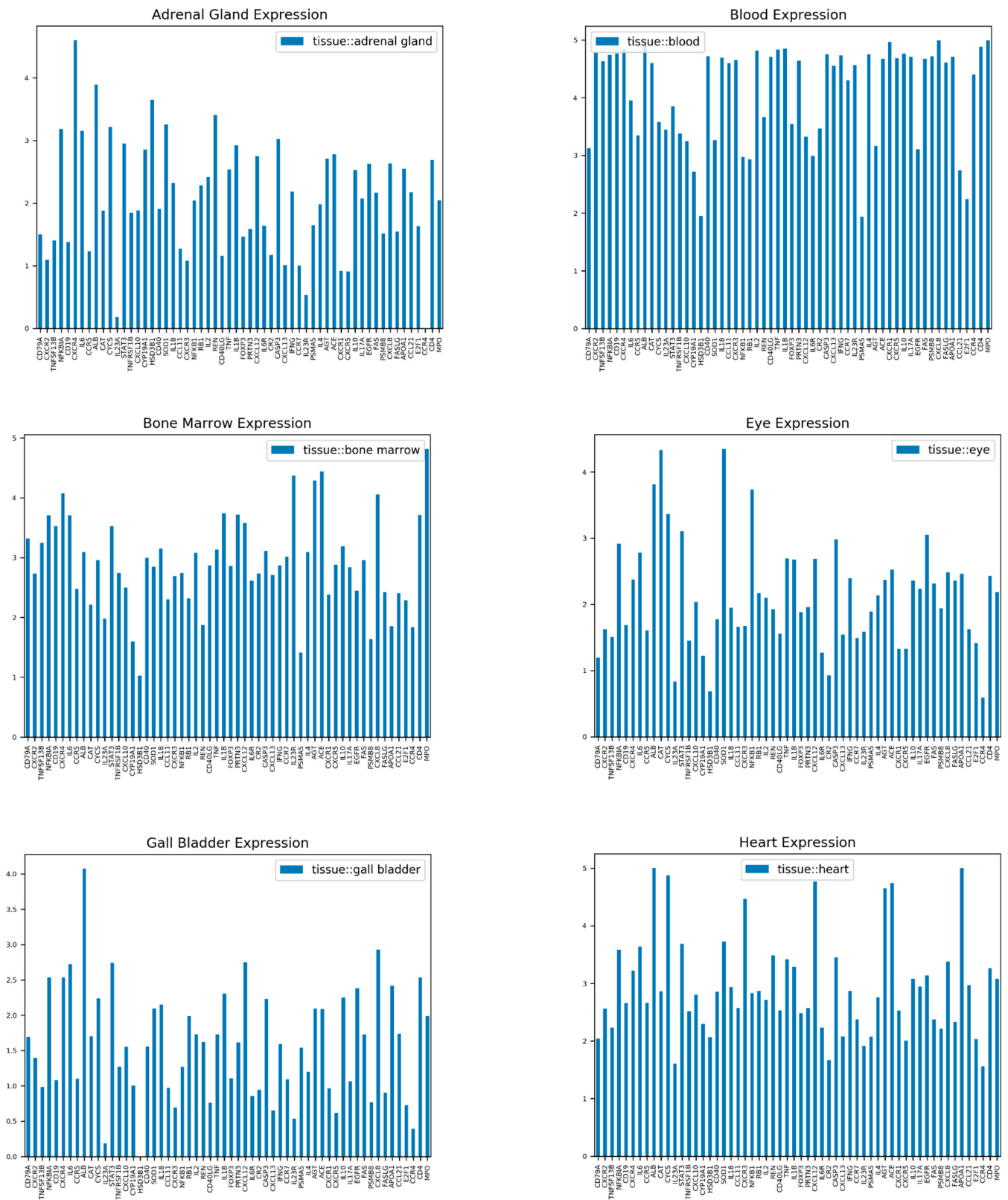

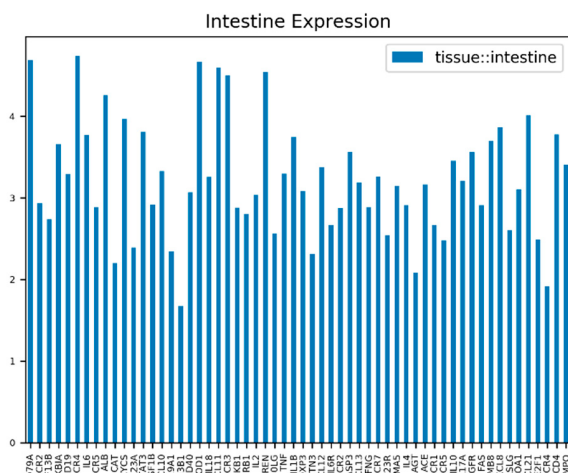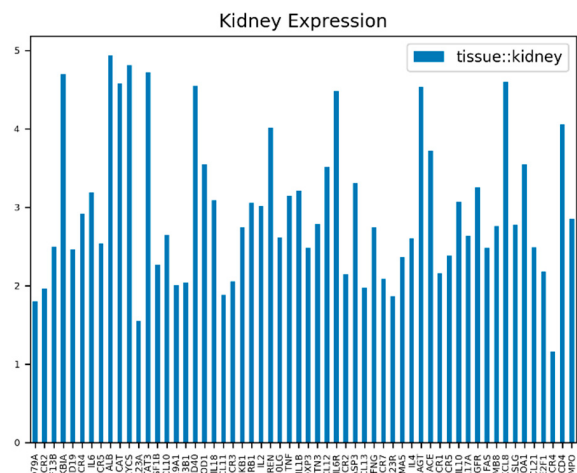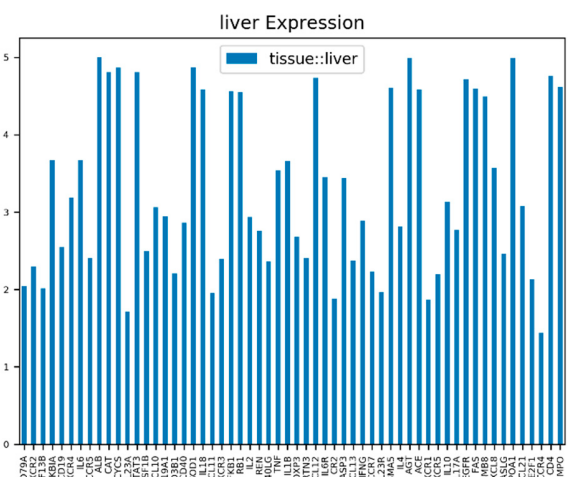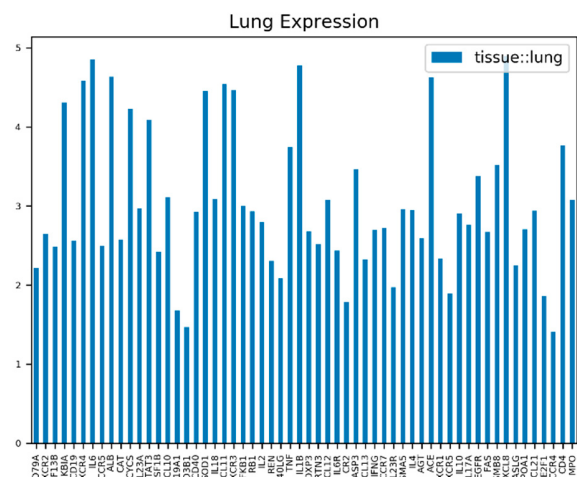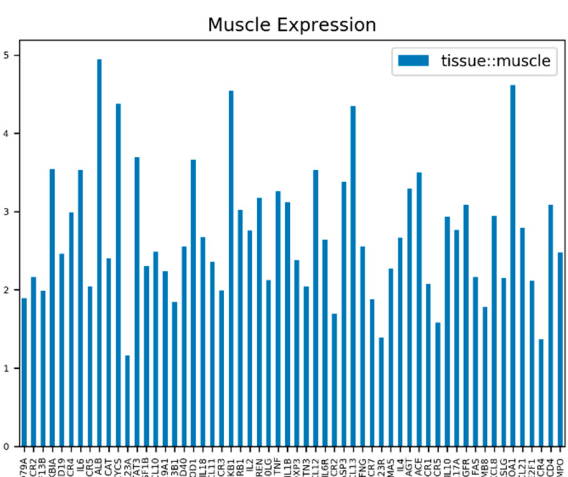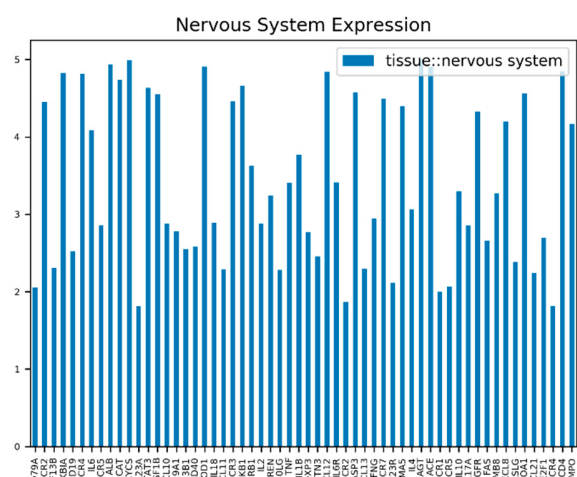

Pancreas Expression

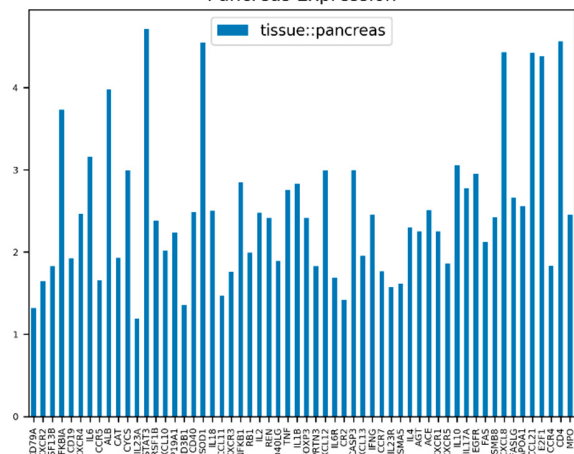

Supplement: Supplementary file 1 [file jcm-11-01442-s001.zip › Figure S3. Cytoscape tissue expression MB 06 09 21.pdf]
